# Investigating the importance of left atrial compliance on fluid dynamics in a novel mock circulatory loop

**DOI:** 10.1038/s41598-024-52327-6

**Published:** 2024-01-22

**Authors:** Masoud Meskin, Philip Alexander Starkey, Alexander Emil Kaspersen, Steffen Ringgaard, Signe Gram Sand, Jens Vinge Nygaard, Jørgen Arendt Jensen, Marie Sand Traberg, Peter Johansen

**Affiliations:** 1 Cardiovascular Biomechanics Group, Department of Health Technology, Technical University of Denmark, Kgs. Lyngby, Denmark; 2 Cardiovascular Experimental Laboratory, Department of Electrical and Computer Engineering, Aarhus University, Finlandsgade 22, 8200 Aarhus N, Denmark; 3 Department of Cardiothoracic and Vascular Surgery, Aarhus University Hospital, Aarhus, Denmark; 4 MR Research Centre, Aarhus University, Aarhus, Denmark; 5 Biomechanics and Mechanobiology, Department of Biological and Chemical Engineering, Aarhus University, Aarhus, Denmark; 6 Center for Fast Ultrasound Imaging, Department of Health Technology, Technical University of Denmark, Kgs. Lyngby, Denmark

**Keywords:** Biomedical engineering, Cardiology

## Abstract

The left atrium (LA) hemodynamic indices hold prognostic value in various cardiac diseases and disorders. To understand the mechanisms of these conditions and to assess the performance of cardiac devices and interventions, in vitro models can be used to replicate the complex physiological interplay between the pulmonary veins, LA, and left ventricle. In this study, a comprehensive and adaptable in vitro model was created. The model includes a flexible LA made from silicone and allows distinct control over the systolic and diastolic functions of both the LA and left ventricle. The LA was mechanically matched with porcine LAs through expansion tests. Fluid dynamic measures were validated against the literature and pulmonary venous flows recorded on five healthy individuals using magnetic resonance flow imaging. Furthermore, the fluid dynamic measures were also used to construct LA pressure–volume loops. The in vitro pressure and flow recordings expressed a high resemblance to physiological waveforms. By decreasing the compliance of the LA, the model behaved realistically, elevating the a- and v-wave peaks of the LA pressure from 12 to 19 mmHg and 22 to 26 mmHg, respectively, while reducing the S/D ratio of the pulmonary venous flowrate from 1.5 to 0.3. This model provides a realistic platform and framework for developing and evaluating left heart procedures and interventions.

## Introduction

The left atrium (LA) plays a pivotal role in the normal functioning of the heart. Traditionally, the LA has been viewed primarily as a chamber that passively transports blood from the pulmonary veins (PVs) into the left ventricle (LV) when the mitral valve (MV) opens. However, the role of LA hemodynamics in various cardiovascular conditions, including heart failure, atrial fibrillation, pulmonary hypertension, and MV dysfunction, is well-recognized^1–10^. LA hemodynamic measures like volume, pressure, and flow provide important insights into cardiac function and have prognostic value in the evaluation of cardiovascular diseases^11–25^.

The LA has three distinct phases of function during the cardiac cycle. In the first phase, it acts as a reservoir for blood flow from the PVs during LV systole after MV closure. In the second phase, it serves as a conduit for passive blood flow from the PVs and LA into the LV during LV early diastole upon MV opening. In the third phase, it actively pumps blood into the LV during late LV diastole. The conduit and pumping phases contribute to LV diastolic filling and preload, where the LA contraction increases stroke volume by 15–30%^3,4^.

**Figure 1:**
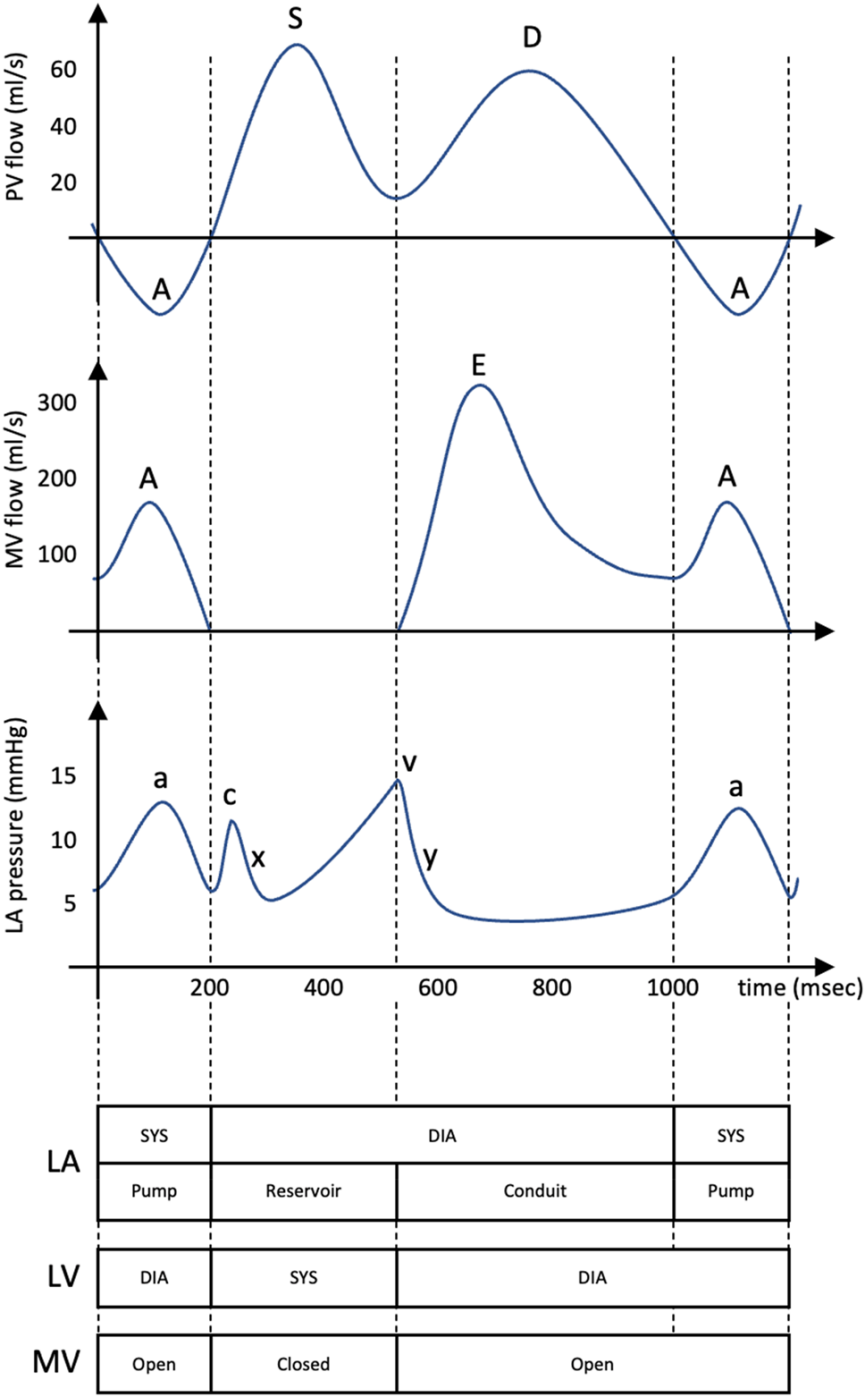
The relationship between various landmarks in the pulmonary venous flow, the mitral valve flow, and the left atrial pressure. These waveforms are related to the left atrial and left ventricular phases of the cardiac cycle, as well as the state of the mitral valve. *DIA* diastolic phase, *LA* left atrium/atrial, *LV* left ventricle/ventricular, *MV* mitral valve, *PV* pulmonary vein/venous, *SYS* systolic phase.

During the cardiac cycle, specific features can therefore be observed in the waveforms of the PV and MV flow and the LA pressure as schematically depicted in Fig. 1. Atrial contraction (the atrial pumping phase) is accompanied by an increase in LA pressure (the a-wave), retrograde flow in the PVs (the A-wave), and an increase in MV flow as a peak (also referred to as the A-wave). As LV contraction begins (the isovolumetric contraction phase), the MV closes and protrudes into the LA, leading to a small rise in the LA pressure (the c-wave). After the c-wave, LA relaxation causes a decrease in the LA pressure (the x-descent). During LV contraction (the atrial reservoir phase), LA pressure increases due to passive filling of the LA (the v-wave), which is caused by PV systolic forward flow reflected as a peak in PV flow (the S-wave). The PV systolic forward flow during the passive filling of the LA is influenced by the relaxation and compliance of the LA and is enhanced by the drop in LA pressure following LA contraction. At the start of LV diastole, the MV opens (beginning of the atrial conduit phase), leading to a rapid filling of the LV (the E-wave peak in the MV flow) and a decrease in LA pressure (the y-descent). At the same time a peak occurs in the PV flow (the D-wave). These detailed hemodynamics features reflect the normal function and intricate interaction between the PV, LA, MV, and LV.

**Figure 2:**
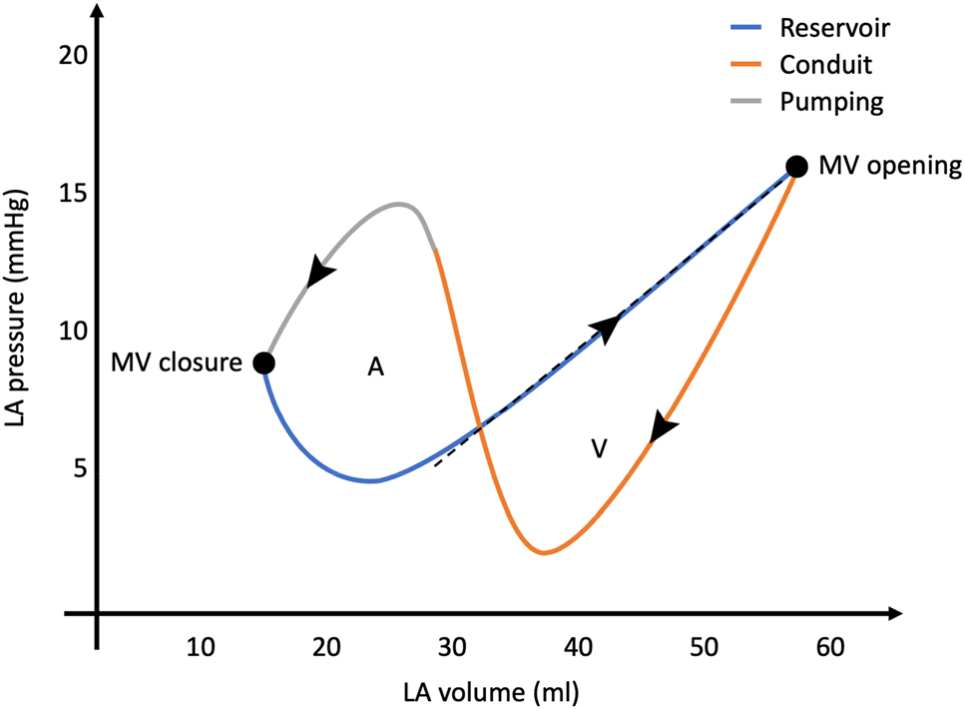
A typical representation of a left atrial pressure–volume loop. The arrows indicate the time course. Mitral valve opening and closure are identified by dots. “A” is the A loop representing the active phase of the atrial cycle and “V” is the V loop associated to the passive phase of the atrial cycle. The blue segment is the reservoir phase; the orange segment is the conduit phase; the grey segment is the pumping phase. The inverse slope of the dashed line represents the atrial compliance. *LA* left atrium/atrial, *MV* mitral valve.

The LA function can also be is assessed through the analysis of the pressure–volume relationship^1,4,26,27^. Figure 2 illustrates a typical pressure–volume loop of the LA. The A loop corresponds to the active phase of the LA and the V loop corresponds to the passive phase. The stroke work of each phase can be determined as the area enclosed by the respective loops. The stroke volume is the change in volume during the pumping phase, and the compliance of the LA can be evaluated in the passive reservoir phase as the ratio between the change in volume to change in pressure (∆V/∆P), represented as the inverse of the slope of the dashed line in Fig. 2.

To improve and develop treatments and devices based on experimental models of the cardiovascular system, it is important to consider these complex interactions. Examples of such interventional related treatments and devices could be MV repair, annuloplasty, and replacement, as well as LA appendage occlusion in both persistent and paroxysmal atrial fibrillation. Moreover, being able to control and alter both the LA and LV diastolic and systolic function enable simulations of various pathological conditions seen in for example cardiomyopathies and heart failure conditions. The most realistic human cardiovascular models are animal in vivo models. However, for animal ethics the guiding principles are to replace, reduce, and refine the experiments (the three R’s)^28^. An in vitro mock circulatory loop (MCL) provides a controlled and reproducible platform for studying these interactions as an alternative to in vivo experiments^29–32^. Nevertheless, replicating the complex hemodynamics and biomechanics of the LA within a MCL is challenging. Previous MCL studies using a porous media^33–37^, a rigid reservoir^31,32,38–44^, or an elastic sphere to represent the LA^45,46^ have not accurately simulated the three phases of the atrial cardiac cycle. Some researchers have attempted to use a biological native LA in a MCL to preserve the geometry^47,48^, but was unable to replicate the active function of the LA. More recent MCLs have included a silicone-cast LV with dual chamber control^49^, and an anatomically-shaped LA chamber with proper active–passive function^50,51^, but achieving a LA with physiological compliance has proven difficult. Hence, there is still a need for a holistic model that can achieve a realistic active and passive function of the LA with a physiological compliance. Therefore, the aim of this study was to develop a versatile left heart MCL including a LA with the capacity to reproduce both the active and passive physiological functions of the native LA hemodynamically and biomechanically.

## Materials and methods

This study was conducted at the Cardiovascular Experimental Laboratory (CAVE Lab), Department of Electrical and Computer Engineering, Aarhus University, Aarhus, Denmark. Magnetic resonance flow imaging was performed at the MR Research Centre, Aarhus University Hospital, Aarhus, Denmark.

The study consisted of several sub-studies to achieve the overall aim. It was important to select a material with mechanical properties similar to those of the native LA tissue to replicate the passive function of the LA. As a benchmark, compliance tests of porcine LA were conducted. Additionally, a thorough understanding of the inlet flow from the PVs was necessary to accurately replicate the LA preload. As a reference for this, pulmonary venous flow recordings using magnetic resonance imaging on healthy persons were performed. The control of the MCL also required timely pressurization of the LA to produce its active function. Both the active and passive LA functions relied on the LV diastolic and systolic function, which was achieved by controlling the LV volume over time.

To complete the MCL with the systemic afterload, peripheral units needed to accurately mimic systemic vascular resistance and compliance.

### Design and construction of the artificial left atrium

**Figure 3:**
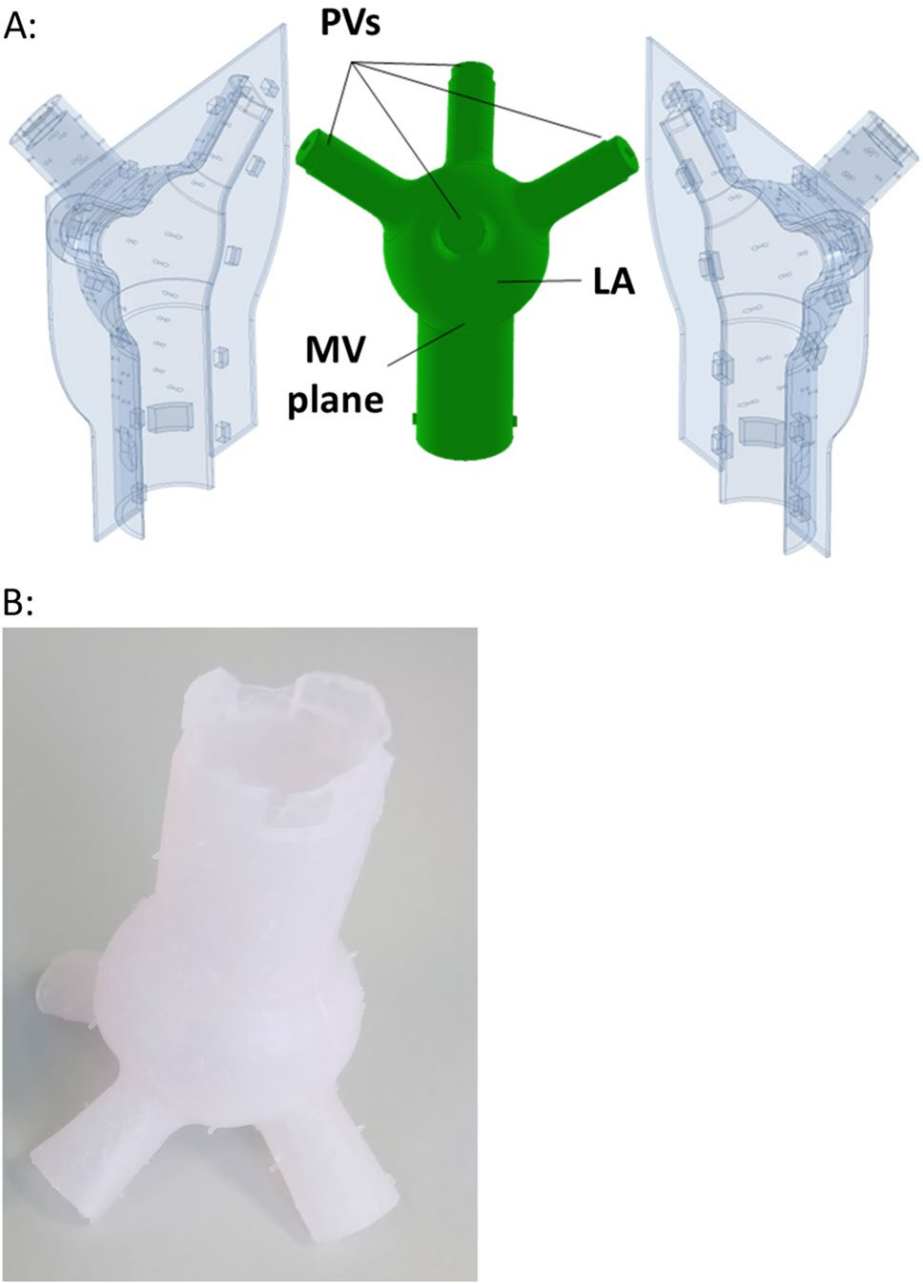
(**A**) Mold for casting of the artificial left atrium, including the outer elastic transparent mold and the inner water-soluble wax core. (**B**) The artificial left atrial chamber.

The artificial LA wall had a thickness of 2 mm^52^, and the radius of the sphere was calculated using the end-systolic volume of the native LA^53^. The radius of the PVs was 6.6 mm, based on the average area of the PVs ostia^54^. The MV annulus had a radius of 25 mm to accommodate the installation of a St. Jude Medical mechanical MV in the MCL. To simplify the process of creating an artificial LA for the MCL, the geometry of the native LA was modified. The resulting model was a smooth, symmetrical sphere with four cylindrical inlets representing the PVs and a circular outlet representing the MV outflow (Fig. 3). It did not include the atrial appendage found in the native LA. The model was designed using Autodesk Inventor^®^ software (Autodesk, Inc., California, USA).

The artificial LA chamber was constructed using a combination of gravity casting and an elastic mold (Fig. 3). The casting process was completed in two stages. First, a water-soluble core was produced using Ferris 2283-A Green soluble wax (Freeman Manufacturing and Supply Company, Avon, Ohio, USA) and a silicone mold. Then, the artificial LA was cast using the water-soluble core and an outer elastic mold. The elastic mold was designed using Autodesk Inventor and 3D-printed with Formlabs elastic 50A transparent resin (Formlabs Inc., Somerville, Massachusetts, USA). The compliance of the chamber was achieved by mixing Silicone rubber ZA-SFX-0020 with platinum catalyst and Elite Double 8 (Zhermack SpA, Rome, Italy) in different proportions, ranging from 50 to 95% Silicone rubber and 50–5% Elite Double 8. A total of 11 chambers (LA1–11) were cast.

### Isolation of the left atrium from porcine hearts

To establish a baseline of LA compliance, nine fresh hearts from approximately 80 kg pigs were stored cold and dissected to isolate the LA. The left side of the heart was first separated from the right side. An incision was made horizontally about 1 cm above the apex to provide visual guidance and locate where the right ventricular wall meets the interventricular septum. The right ventricle was then opened with an incision in the anterior wall, running from the cross-sectional opening along the anterior interventricular sulcus to the coronary sulcus. The aortic arch was removed from the ascending aorta by a cross-sectional cut about 3 cm above the aortic annulus. The LA was then separated from the right atrium with two incisions: one in the anterior wall of the right atrium from the coronary sulcus through the pulmonary trunk close to the left leaflet of the pulmonary valve, and the other in the posterior wall from the transverse pericardial sinus to the coronary sinus. The right atrium and ventricle were separated from the left side by an incision from the coronary sinus following the posterior interventricular sulcus. An incision was made vertically in the interventricular septum to access the MV. Both papillary muscles were isolated from the myocardium, the anterior MV leaflet was sutured above the aortic ostium to prevent leakage, while the posterior leaflet was sutured to the LV wall to keep the MV open. The PVs were sutured to completely seal the LA inlets.

### Compliance tests

#### Compliance test setup

**Figure 4:**
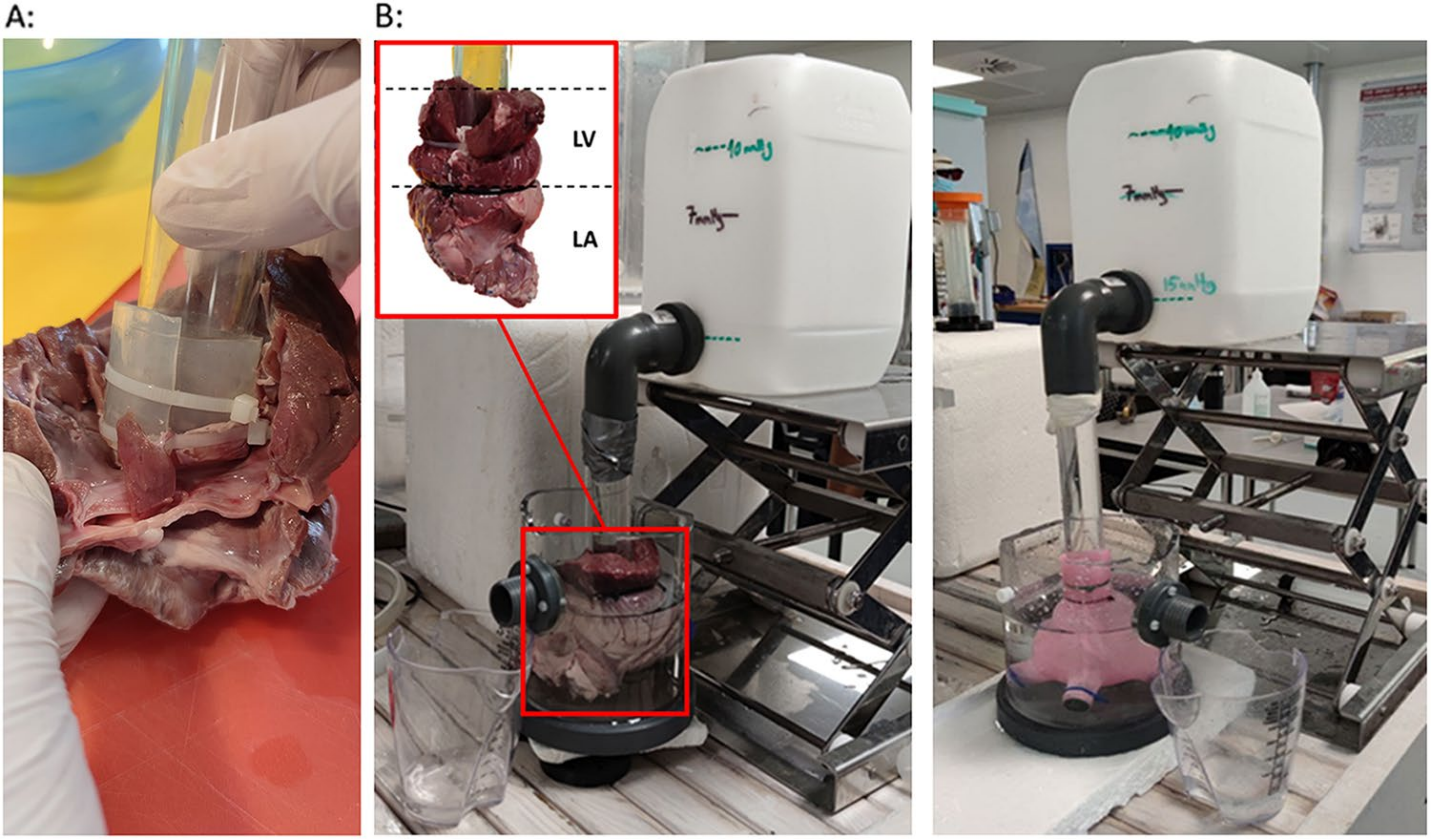
(**A**) Picture of the polycarbonate tube installed in the isolated left atrium. (**B**) The experimental setup for the expansion measurements on both the artificial and the native left atrial chambers.

The LA was installed in an experimental setup (Fig. 4) that included a transparent polycarbonate tube (diameter 25 mm) with a straight end placed inside the LV, ensuring that the tip of the tube was aligned with the edges of the MV annulus. This initial step involved the precise placement of the tip of the tube at the MV ostium (Fig. 4A). To prevent any unintended movement of the porcine LA from the tip of the tube, a piece of silicone tubing was wrapped around the tip of the tube, enhancing the friction between the porcine LA and the tube. Additionally, during the dissection process, the LV chamber was not entirely removed; a portion of the LV was intentionally retained to facilitate secure attachment of the LA chamber to the tube by wrapping the LV around the tube and securing the position using cable ties. After ensuring that the porcine LA was properly sealed, it was held upside down and filled with water to the MV ostium using a syringe, and the water volume was recorded as a measure of the unpressurized LA volume. This pressure was selected as the 0 Pa reference pressure. To measure the expansion during pressurization a 90-degree elbow joint tube was connecting the other end of the polycarbonate tube to a transparent bucket serving as a water container (Fig. 4B). The water level could be adjusted to provide a hydrostatic pressure that caused the expansion of the LA. The LA was completely submerged in another container filled with water that had an overflow outlet.

#### Compliance test protocol

The LA was pressurized to 2000 Pa (approximately 15 mmHg), which has been reported as the average pressure of the porcine LA during the reservoir phase^17^. When the LA expanded, it caused an equivalent amount of water to be displaced through the overflow outlet and into a measuring cup. The volume of the water collected in the measuring cup was recorded as the volume difference between the unpressurized and pressurized state. The measurements were repeated three times for each LA chamber, and the average volume difference was calculated. The expansion was then calculated as the ratio between the average volume difference and the unpressurized volume in percentage.

The expansion of the artificial LAs was measured using the same method as for the native porcine LAs. The artificial LAs were grouped into three categories when compared to the native LAs, depending on whether the expansion was: (1) Within the native LA range (normal compliance), (2) Below the native LA range (low-compliance), or (3) Above the native LA range (high-compliance). Four of the artificial LAs were selected for the experiments to provide a representation of the different compliances.

### Fluid dynamic tests

#### Reference pulmonary venous flow assessment using magnetic resonance imaging

To assess the reference PV flow profiles, in-vivo 2D phase-contrast magnetic resonance flow imaging was performed in five healthy young volunteers (four males and one female, aged 23–33 years). All participants gave informed consent and the study was approved by the local ethics committee (Central Denmark Region, Denmark). All experiments were performed in accordance with relevant guidelines and regulations. The imaging was performed using a Philips magnetic resonance scanner (Achieva dStream 1.5T, Philips, Best, Netherlands). Initially, a multi-slice, multi-phase Balanced-Steady-State-Free-Precession sequence was applied over the entire heart to locate the position and orientation of the vessels. The flow in the four PVs was measured proximal to the PVs ostium over 40 cardiac phases. The slice thickness was 8 mm, and the pixel size was 1.2 × 1.2 mm. The flow rate of each PV was measured in a slice orthogonal to and 3 mm proximal to the PV ostium. The images were analyzed using in-house written software.

#### Fluid dynamic in vitro test setup

**Figure 5:**
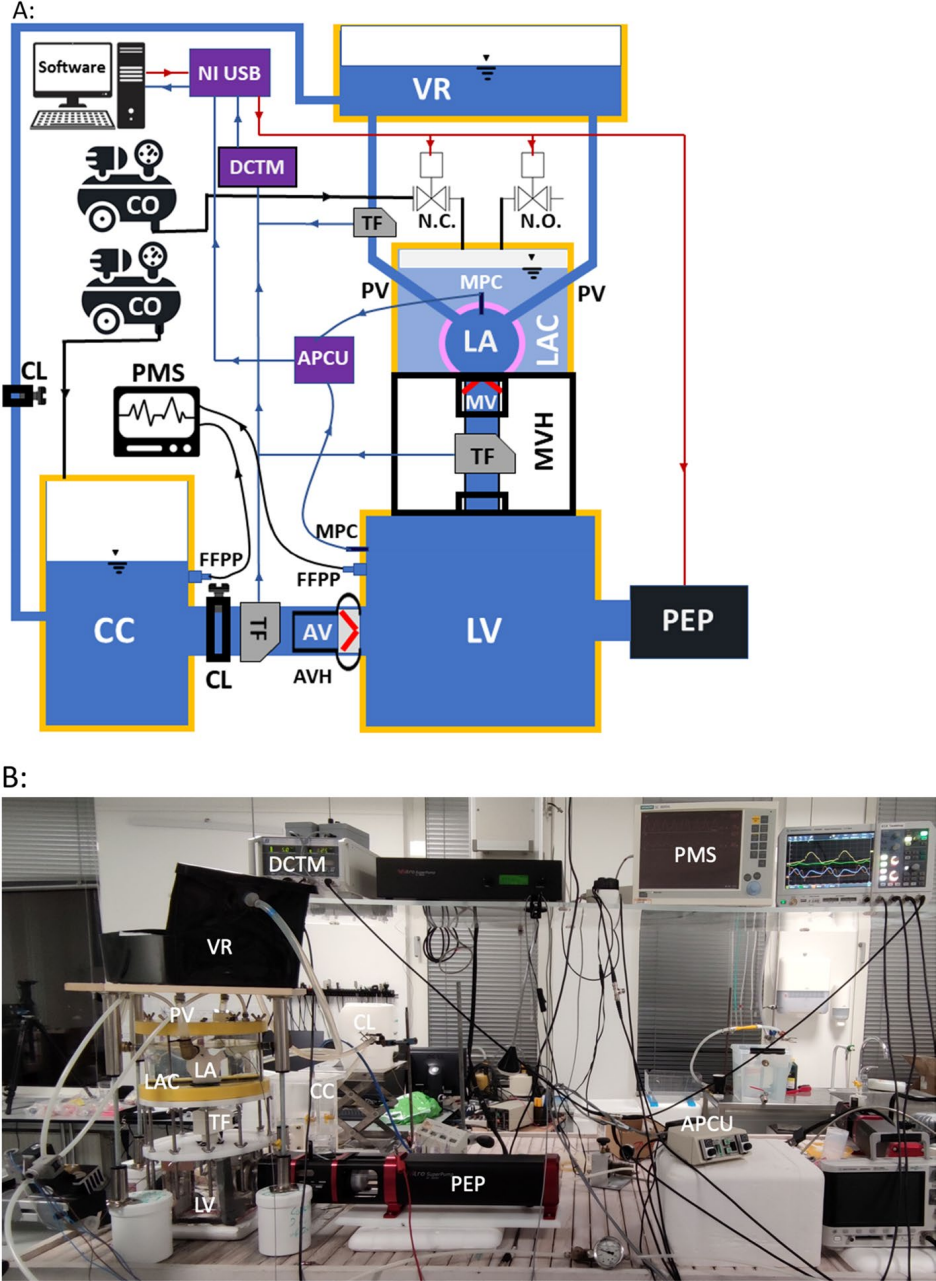
(**A**) Schematic representation of the mock circulatory loop setup. (**B**) Image of the mock circulatory loop. Hydraulic part elements (following flow direction): *PEP* pulsatile electromechanical pump, *LV* left ventricular chamber, *AV* aortic valve, *AVH* aortic valve housing, *CL* clamp, *CC* compliance chamber, *VR* venous reservoir, *PV* pulmonary vein, *LAC* left atrial container, *LA* artificial left atrial chamber, *MV* mitral valve, *MVH* mitral valve housing. Elements devoted to control and measurements: *APCU* amplifier pressure control unit, *CO* compressor, *DCTM* dual channel tubing module, *FFPP* fluid filled pressure probes, *MPC* micro-tip pressure catheter, *N.C.* normally closed valve, *NI USB* data acquisition board, *N.O.* normally open valve, *PMS* patient monitoring screen, *TF* tubing flow sensor. The red arrow lines indicate the generated triggering signal from the software, controlling the pulsatile electromechanical pump piston head, and the normally-closed and the normally-open valves controlling the pressurization of the left atrial container. The blue arrow lines are the measured pressure and flow data, sent to the data acquisition software.

The MCL model in Fig. 5 is an improved version of a previous in-vitro model of the left heart^31,32,55^. It includes a pulsatile electromechanical pump (SuperPump AR Series, ViVitro Labs, Victoria, British Columbia, Canada) that provides the volume changes in the rigid LV chamber during the cardiac cycle. The outlet of the LV chamber is through an aortic valve mounted in a rigid aortic root, which is connected to a clamped silicone tube simulating the aorta. The aorta is connected to a compliance chamber that simulates arterial compliance, which is connected to a venous reservoir through a clamped silicone tube controlling the systemic resistance. The primary controllable components of the systemic afterload are therefore the compliance chamber (directly affecting the pulse pressure and waveform shape) and the two clamps controlling the systemic resistance (directly affecting the systemic pressure). The venous reservoir is connected to the LA through four silicone tubing PVs and has a large capacity, providing an almost static PV pressure head despite a slightly pulsatile inlet flow. The LA is embedded in a container, which is pressurized and vented using solenoid valves (type EV210A, Danfoss A/S, Nordborg, Denmark) to replicate LA pulsatility. Flow rates through the PVs, MV, and aorta are measured using tubing flow sensors connected to a flowmeter module (PXL11, PXL25, TS410, Transonic Systems Inc.^®^, Ithaca, New York, USA), and pressure in the LA and LV is recorded using fluid-filled pressure catheters and micro-tip pressure catheters connected to a pressure control unit (SPC-350MR, PCU-2000, Millar Inc., Houston, Texas, USA). The working fluid in the model is distilled water.

To operate, coordinate and synchronize the functions of the MCL components, a custom software program was developed using LabVIEW (National Instruments, Austin, Texas, USA), which allows the operator to control the pump and solenoid valves and collect data simultaneously. The software generates a LV volumetric waveform, which controls the piston position of the pump, and it also provides the signal to control the diastolic and systolic phases of the LA by triggering the solenoid valves at an output sample rate of 1 kHz. Additionally, the software handles calibration, display, and recording of flow rates and pressure data at a sampling rate of 1 kHz using a NI USB-6259 module (National Instruments, Austin, Texas, USA). The LV volumetric waveform consists of three separate cosine waveforms that represent the three cardiac phases: systole, early diastole, and late diastole. These waveforms are described by the following equations:

1 $${\text{Systole}}:\quad f(t) = a_{sys} \cdot \cos \left( {t \cdot \frac{\pi }{{t_{sys} }}} \right)$$

2 $${\text{Early}}\;{\text{diastole}}:\quad f(t) = - a_{ed} \cdot \cos \left( {\left( {t - t_{sys} } \right) \cdot \frac{\pi }{{t_{ed} }}} \right) - a_{sys} + a_{ed}$$

3 $${\text{Late}}\;{\text{diastole:}}\quad f(t) = - a_{ld} \cdot \cos \left( {\left( {t - \left( {t_{sys} + t_{ed} } \right)} \right) \cdot \frac{\pi }{{t_{ld} }}} \right) - a_{sys} + 2a_{ed} + a_{ld}$$

In these equations, *t* is the time, and $t_{sys}$, $t_{ed}$, $t_{ld}$ are the systolic, early-diastolic, and late-diastolic time spans, respectively. The coefficients $a_{sys}$, $a_{ed}$, $a_{ld}$ are the amplitudes of the corresponding waveforms and are related by Eq. (4).

4 $$- a_{sys} + a_{ed} + a_{ld} = 0$$

**Figure 6:**
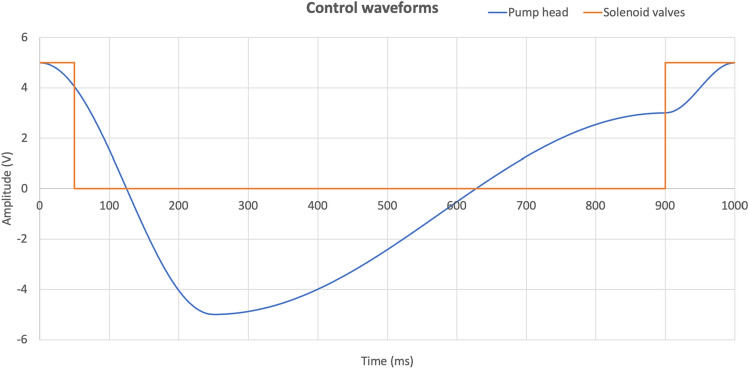
An example of the control waveforms for the piston pump head position (blue) and solenoid valves (orange). The piston moves forward when the slope of the control signal is negative. The LA container is pressurized when the solenoid control signal is high (5 V) and vented when low (0 V).

Equation (4) ensures that the LV volumetric waveform starts and ends at the same voltage level. The time span and amplitude of each cosine waveform can be modified to simulate various clinical cardiac scenarios. The trigger signal for the solenoid valves is the same for both pressurization (normally-open) and venting (normally-closed) operations. Figure 6 depicts these control waveforms for a cycle time of 1 s, corresponding to a heart rate at 60 beats per minute.

The procedure for configuring the flow loop to achieve the desired target conditions begins with adjusting the stroke volume of the piston pump to regulate the cardiac output. Following this, optimization of the target pressure and pressure waveform shape is accomplished by fine-tuning the mean pressure through adjusting the resistance clamps and the pulse pressure through the compliance chamber. The fine-tuning of the air volume in the compliance chamber is carried out using manual ball valves that facilitate venting and pressurization. The pressure source is a compressor operating at 1 atm. However, the valves are only very gently adjusted until the pressure waveform of the compliance chamber exhibits the desired physiological characteristics. Similarly, the pressure supplied to the LA container is fine-tuned until the desired PV flow and LA pressure is achieved.

#### Fluid dynamic test protocol

Fluid dynamics measurements using the MCL were conducted after installing the artificial LA chamber in the model. The duration of each cardiac phase and the cardiac output were adjusted by manipulating the period and amplitude of the three cosine waveforms. The systolic time ($t_{sys}$) was set to 350 ms, early diastole ($t_{ed}$) to 500 ms, and late diastole ($t_{ld}$) to 150 ms. A cardiac output of 4.5 l/min was achieved by adjusting the gain on the ViVitro pump controller. The MCL was initially run for several cycles to remove air bubbles from the system.

#### Fluid dynamic data analysis

Twenty heart cycles of pressure and flow raw data were acquired. Ensemble averaging with standard deviation was calculated, and from this the following parameters were deduced: LA pressure (a-wave peak; v-wave peak; mean value; minimum value), MV flow (E-wave peak; A-wave peak; E/A ratio), PV flow (A-wave peak; S-wave peak; D-wave peak; S/D ratio).

The time average of the standard deviation over the cardiac cycle was calculated as a measure of the beat-to-beat variation.

The ensemble averaged pressure and flow data are used to generate the pressure–volume loops of the artificial LA1, LA5, LA10, and LA11. The LA volume as a function of time is not measured directly but estimated based on the measured inlet flow on one of the PVs and the MV outlet flow. Assuming continuity and no leakage the volume entering the LA must equal the volume leaving the LA over one cardiac cycle. The relative inlet and outlet volumes are calculated as the accumulation of the recorded MV and PV flow (Eq. (5)).

5 $$V_{R} [n] = \frac{1}{{f_{s} }}\mathop \sum \limits_{i = 0}^{n} Q[i]$$

$V_{R} [n]$ are the relative volume sample values, $Q[i]$ is the sampled flow rate, and the sampling frequency *f*_*s*_ = 1000 Hz.

As the inlet flow is only measured in one of the four PV’s, the total PV volume is scaled relative to the MV volume to ensure continuity. The total LA volume function, *V*_*LA*_, over the cardiac cycle is then estimated as

6 $$V_{LA} [n] = \left( {V_{R,PV} [n] - V_{R,MV} [n]} \right) + V_{0}$$

where $V_{R,PV} [n]$ is the scaled relative PV inlet volume function, $V_{R,MV} [n]$ is the relative MV outlet volume function, and $V_{0}$ is the initial volume which is assumed to be equal the volume of the artificial LA in its unstressed state. $V_{0}$ is calculated based on the computer aided design model of the LA to be 67 ml.

To reduce noise and oscillations from the recorded pressure in the constructed pressure–volume loops the LA pressure is filtered using a centralized moving average filter with equal coefficients:

7 $$P_{filt} [i] = \frac{1}{M}\mathop \sum \limits_{{j = - \frac{M}{2}}}^{{\frac{M}{2} - 1}} P_{rec} [i + j]$$

where $P_{rec}$ is the recorded pressure, and $P_{filt}$ is the filtered pressure. The filter order *M* = 150.

On the pressure–volume loops the MV opening and the MV closure will be indicated based on the start of the E-wave on the MV flow curve, and at the zero-crossing at the end of the A wave on the MV flow curve, respectively. This will also together with the turn point from the end of the E-wave to the start of the A-wave on the MV flow curve be used to identify the reservoir, conduit, and pumping phases on the pressure–volume loops.

#### A and V loop stroke work in the left atrial pressure–volume loop

From the LA pressure–volume loop the area of the A loop (representing the active phase of the atrial cycle) and the V loop (representing the passive phase of the atrial cycle) are calculated as a measure of stroke work. Each of the A and V loops are segmented from the entire pressure–volume loop at the crossing point. To calculate the area, Greens theorem is applied in a discrete form using polygonal approximation:

8 $$Area = \left| {\mathop \sum \limits_{i = 0}^{N - 1} \left( {P[i + 1] - P[i]} \right) \cdot \frac{V[i + 1] + V[i]}{2}} \right|$$

where *P* is the LA pressure, *V* is the LA volume, and *N* is the number of samples in the heart cycle. Taking the absolute value ensures that the area becomes positive when going either clockwise or counterclockwise.

The compliance (ΔV/ΔP) of the artificial LAs are estimated as the inverse slope of the reservoir phase where the pressure–volume loop exhibits a linear relationship with a steady volume and pressure increase. A linear fit is used to determine the slope.

The LA stroke volume is determined as the difference in LA volume from the start to the end of the LA systole (pumping phase).

## Results

### Expansion of porcine and artificial left atriums

**Figure 7:**
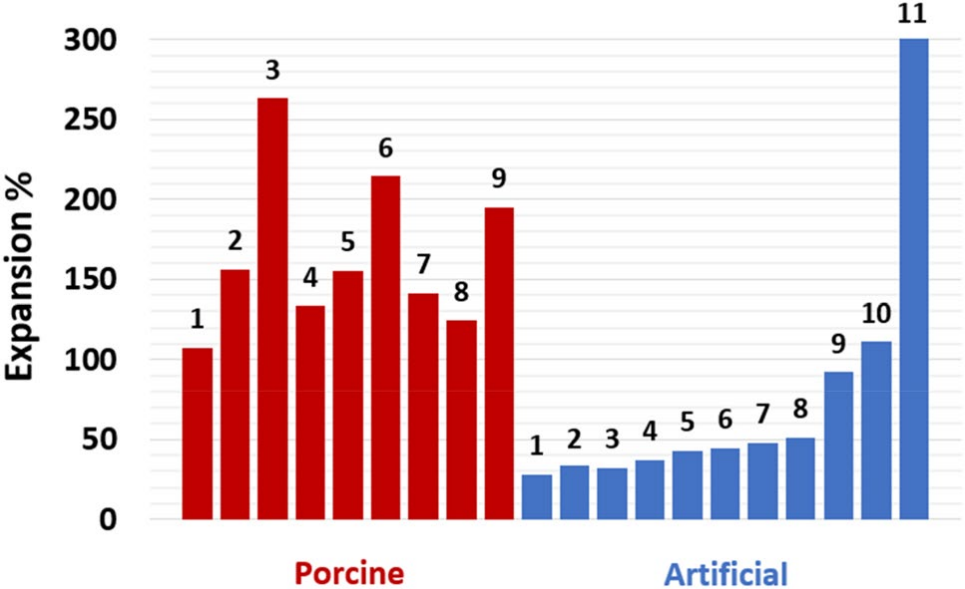
Bar plots of the expansion in percentage of each of the nine native porcine atriums (red bars 1–9) and the eleven artificial left atriums (blue bars 1–11).

The results of the expansion test of the porcine and artificial LAs are reported in Table 1. The expansion percentage of the porcine LAs was in the range 107–263% with a median value of 156%. The lowest and highest expansion was observed in porcine LA1 (107%) and LA3 (263%), respectively. Of the artificial LAs, ten exhibited expansions in the range 28–111%, while one had an expansion of more than 300%. A comparison between the expansions of the native LAs and artificial LAs can be seen in Fig. 7. The artificial LA1–9 were categorized as low-compliance, LA10 as normal-compliance, and LA11 as high-compliance. Amongst the eleven artificial LAs, LA1, LA5, LA10, and LA11 were selected for the experiments.

**Table 1:** Unpressurized volume, pressurized volume, and expansion in percentage of each of the native porcine and artificial left atriums, and the mixture ratio of each artificial left atrium.

LA	Native porcine LAs			Artificial LAs			
	Unpressurized volume (ml)	Pressurized volume (ml)	Expansion (%)	Mixture ratio (%)^a^	Unpressurized volume (ml)	Pressurized volume (ml)	Expansion (%) mean ± SD
1	27	56	107	50/50	67	86	28 ± 1.7
2	32	82	156	55/45	65	78	34 ± 0.0
3	38	138	263	60/40	66	87	32 ± 0.0
4	30	70	133	65/35	66	90	37 ± 0.9
5	45	115	156	70/30	70	100	43 ± 0.0
6	14	44	214	75/25	65	94	45 ± 1.5
7	34	82	141	80/20	68	100	48 ± 0.9
8	37	83	124	85/15	68	103	51 ± 0.8
9	38	112	195	90/10	70	135	92 ± 3.7
10	–	–	–	95/5	69	145	111 ± 3.4
11	–	–	–	100/0	69	> 500	> 300

^a^Mixture ratio of silicone rubber/elite double 8.

*LA* left atrium, *SD* standard deviation.

### Flowrate profiles of the native pulmonary veins

**Figure 8:**
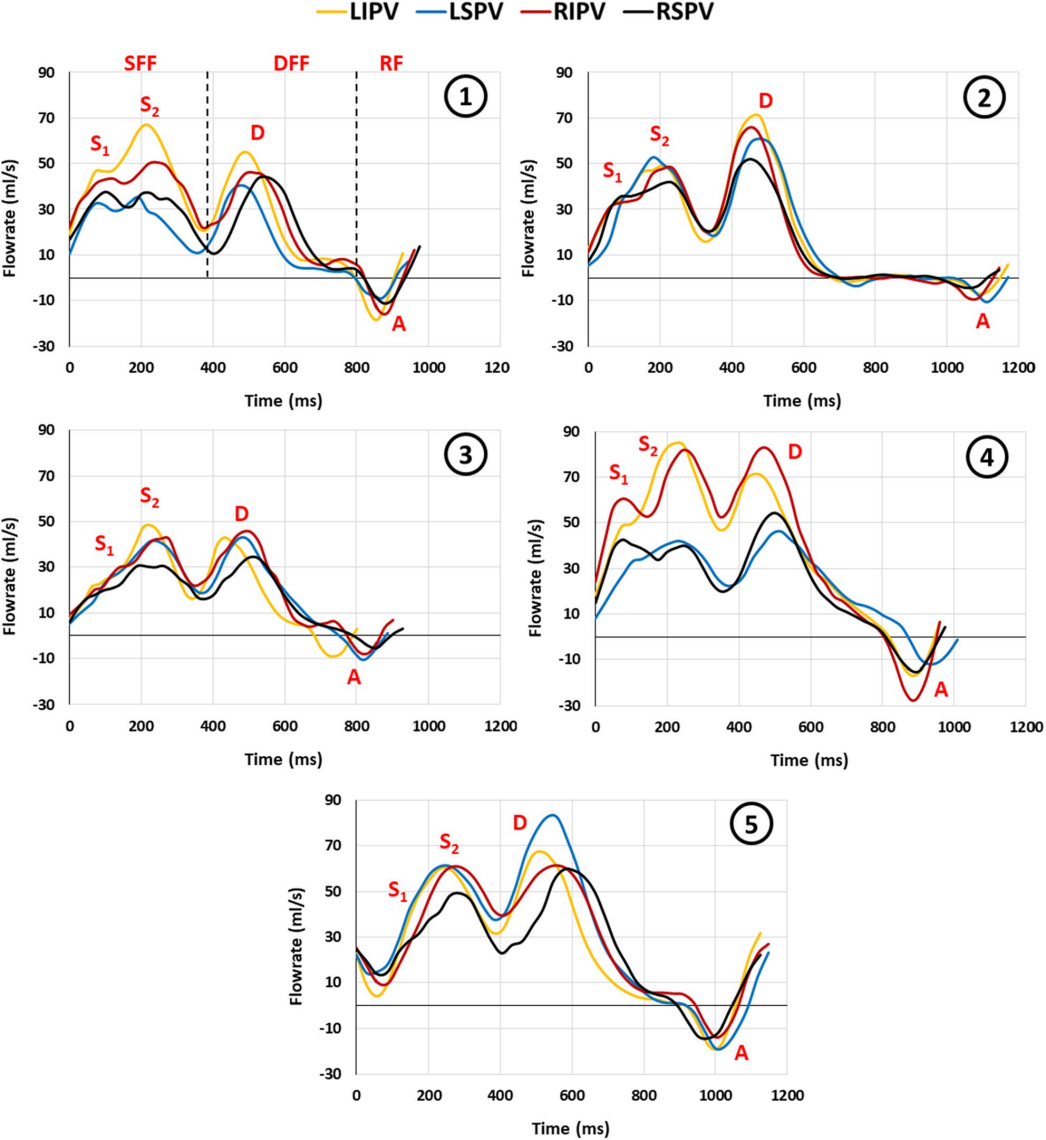
The flowrate profiles of the native pulmonary veins of five healthy young volunteers (panel 1–5). *DFF* diastolic forward flow, *LIPV* left inferior pulmonary vein, *LSPV* left superior pulmonary vein, *RF* retrograde flow, *RIPV* right inferior pulmonary vein, *RSPV* right superior pulmonary vein, *SFF* systolic forward flow.

All five volunteers who underwent 2D phase-contrast magnetic resonance flow imaging had four PVs. The flowrate profiles of the left inferior, left superior, right inferior, and right superior PV for each subject are shown in Fig. 8, where the distinct S-, D-, and A-waves are clearly seen and labeled. The systolic phase had a duration of 350–400 ms for all individuals, while the variation in the diastolic phase was larger due to the different heart rates.

### Fluid dynamic measurements

**Figure 9:**
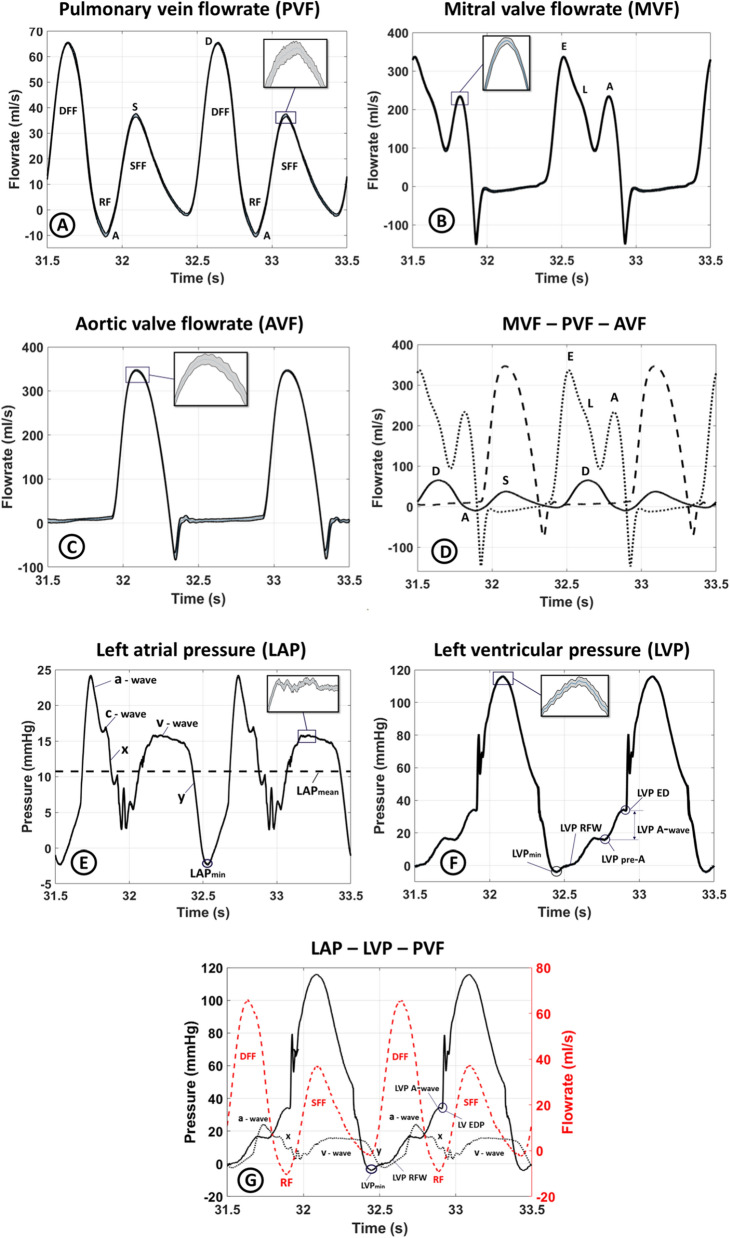
The fluid dynamic measurements with the artificial left atrium 10 with the expansion closest to the native porcine left atrium. The zoomed area shows the mean and the standard deviation in all the graphs. All profiles show two cardiac cycles. (**A**) The pulmonary vein flowrate profile. *DFF* diastolic forward flow, *SFF* systolic forward flow, *RF* retrograde flow, *D* diastolic peak, *S* systolic peak. (**B**) The mitral valve flowrate profile. *E* early diastolic peak, *A* atrial kick. (**C**) The aortic valve flowrate profile. (**D**) The pulmonary vein, mitral valve, and aortic valve flowrate profiles displayed together for easy comparison. (**E**) The left atrial pressure. The a-wave, v-wave, c-wave, and x and y troughs are marked on the graph. The dashed line indicates the mean left atrial pressure. (**F**) The left ventricular pressure. *LVPmin* minimum left ventricular pressure, *LVP-RFW* left ventricular rapid filling wave, *LVP-pre-A* left ventricular pressure prior to the left atrial contraction, *LVP-ED* end-diastolic left ventricular pressure. (**G**) The left atrial pressure (black dotted line), left ventricular pressure (solid black line), and pulmonary vein flowrate (red dashed line) plotted together to show the relation between the left atrial pressure, left ventricular pressure, and pulmonary vein flowrate.

The in vitro MCL flow and pressure results using the artificial LA10, which most closely mimics the native porcine LA, are displayed in Fig. 9. Each waveform is represented as the ensemble average of 20 cycles with the standard deviation. To demonstrate the stability and low beat-to-beat variation, a zoomed panel is provided for each flow and pressure measurements. Moreover, as a measure of repeatability the time average of the standard deviation over the cardiac cycle for each signal for LA1, LA5, LA10, and LA11 is reported in Table 2.

**Table 2:** The time average over the cardiac cycle of the standard deviation as a measure of beat-to-beat variation for the recorded left atrial pressure, left ventricular pressure, mitral valve flow, pulmonary venous flow, and aortic flow for the artificial left atrium number 1, 5, 10, and 11.

LA	LA pressure	MV flow	PV flow	LV pressure	AV flow
1	0.28	3.01	0.95	0.94	4.10
5	0.23	2.30	0.75	0.73	2.61
10	0.14	2.32	0.72	0.61	4.13
11	0.15	2.83	0.67	0.80	4.08

*AV* aortic valve, *LA* left atrium, *LV* left ventricle, *MV* mitral valve, *PV* pulmonary vein.

#### Waveform characteristics

The PV flow profile (Fig. 9A) includes the two antegrade S- and D-waves and the retrograde A-wave. The S- and D-peaks are approximately 37 and 65 ml/s, respectively, resulting in an S/D ratio of 0.57. Figure 9B shows the MV flow, which exhibits distinct E- and A-waves and is near zero during LV systole when the MV is closed. Similarly, the aortic valve flow (Fig. 9C) is close to zero during LV diastole when the aortic valve is closed and peaks during LV systole. Valve closing volumes are seen as short retrograde flows observed for both the MV and aortic valve. Figure 9D provides a temporal comparison of the MV, PV, and aortic valve flow. The LA pressure (Fig. 9E) exhibits the a-, c-, and v-waves along with the x- and y-descents. The a- and v-wave peaks were measured to be approximately 24 and 16 mmHg, respectively. Figure 9F shows the LV pressure, which exhibits a systolic peak of approximately 115 mmHg. The closure of the mechanical MV is seen as the pressure fluctuations during the systolic pressure increase.

#### Different left atrial compliances

**Figure 10:**
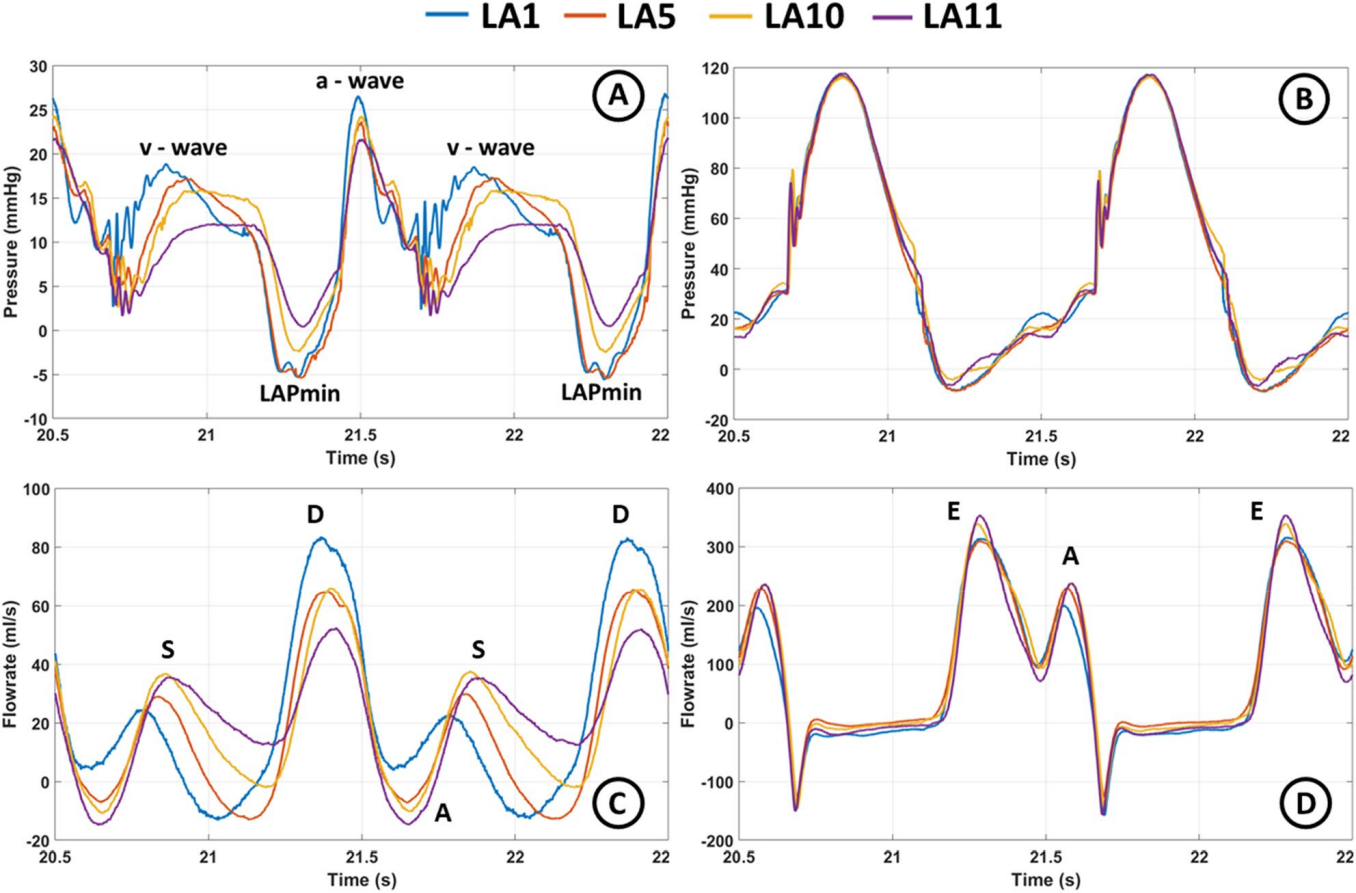
Recorded pressures and flows for the artificial left atrium (LA) 1, 5, 10, and 11. (**A**) Left atrial pressure, (**B**) left ventricular pressure, (**C**) pulmonary venous flow, and (**D**) mitral valve flow. *LA* left atrium, *LAPmin* minimal left atrial pressure.

The pressure and flow data obtained from the MCL fluid dynamics measurements with various levels of LA compliance (LA1, LA5, LA10, and LA11) are illustrated in Fig. 10 and summarized in Table 3. Four different LA chambers were evaluated, including two with low compliance (LA1 and LA5), one with normal compliance (LA10), and one with high compliance chamber (LA11). The data shows a significant impact on the LA pressure as LA compliance decreases (Fig. 10A). The LA v-wave increases from approximately 12 to 19 mmHg going from the highest to the lowest compliance. Moreover, the LA a-wave increases from approximately 22 to 26 mmHg. Figure 10B shows no significant impact on LV pressure. Figure 10C shows a pronounced change in the PV flow due to the decrease in LA compliance, with a decline in the systolic forward flow (the S-wave) and a positive shift of the A-wave. Furthermore, an increase is observed in the diastolic forward flow (the D-wave). This is also reflected in the S/D ratio in Table 3. Figure 10D shows that decreasing the LA compliance also reduces the peak MV flow. The E-wave was reduced from 353 to 313 ml/s and the A-wave was reduced from 234 to 196 ml/s.

**Table 3:** Main features of the recorded left atrial pressure, mitral valve flow, and pulmonary venous flow for the artificial left atrium number 1, 5, 10, and 11.

LA	LA pressure				MV flow			PV flow			
	a-wave (mmHg)	v-wave (mmHg)	Mean (mmHg)	Min (mmHg)	E-wave (ml/s)	A-wave (ml/s)	E/A	A-wave (ml/s)	S-wave (ml/s)	D-wave (ml/s)	S/D
1	18.7	26.4	10.6	− 5.5	313	196	1.6	3.5	23.9	82.6	0.29
5	17.2	23.4	9.5	− 5.4	308	227	1.4	− 6.9	29.2	64.8	0.45
10	15.8	24.1	10.8	− 2.3	337	234	1.4	− 10.1	37.1	65.3	0.57
11	12.0	21.7	9.5	0.4	353	234	1.5	− 14.9	34.9	51.5	1.48

*E/A* E-wave/A-wave, *LA* left atrium/atrial, *MV* mitral valve, *PV* pulmonary vein, *S/D* S-wave/D-wave.

**Figure 11:**
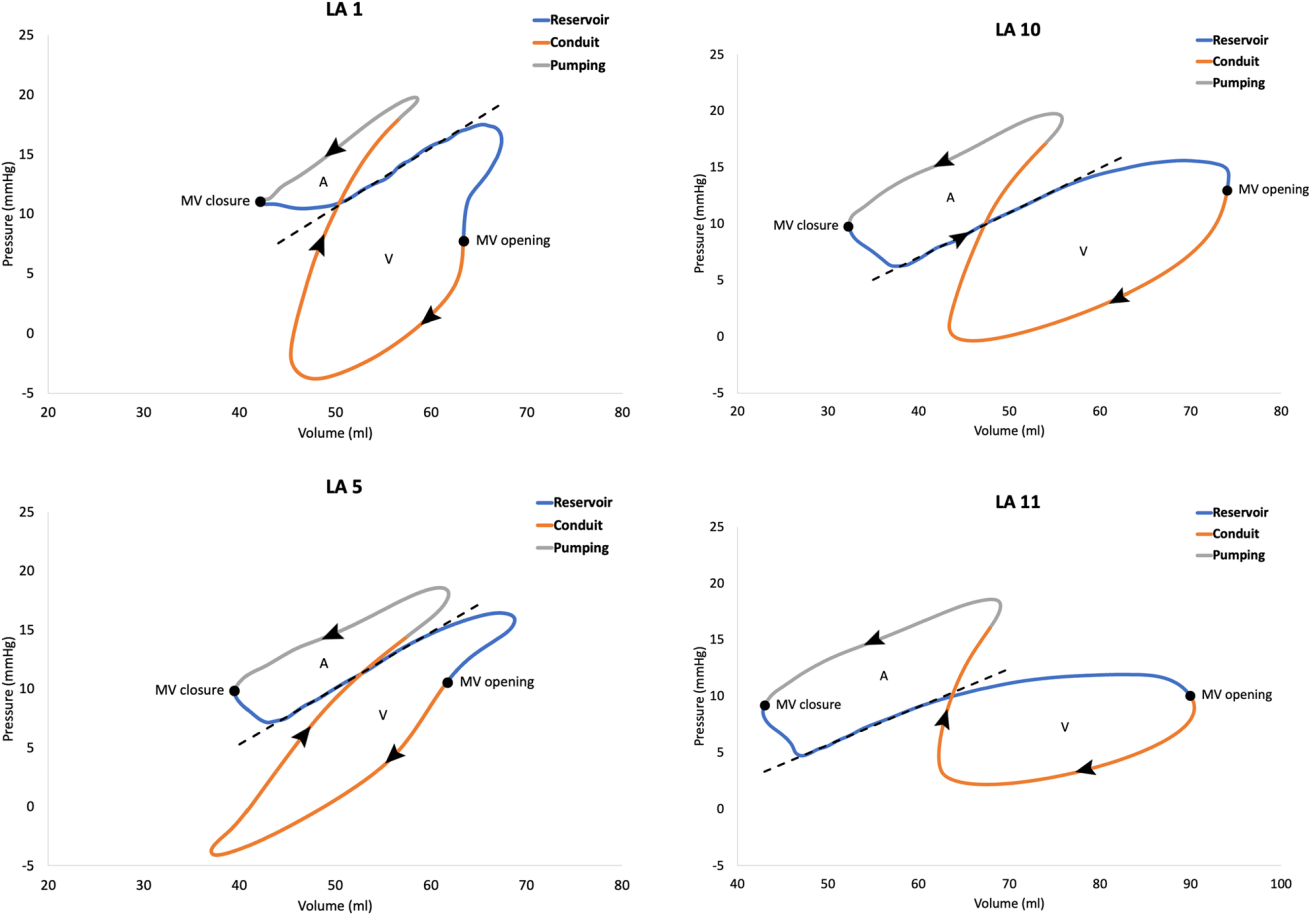
The pressure–volume loop for the artificial left atrium 1, 5, 10, and 11. The arrows indicate the time course. Mitral valve opening and closure are identified by dots. “A” is the A loop representing the active phase of the atrial cycle and “V” is the V loop associated to the passive phase of the atrial cycle. The blue segment is the reservoir phase; the orange segment is the conduit phase; the grey segment is the pumping phase. Note that the volume axis for LA11 is shifted compared to LA1, LA5, and LA10. The inverse slope of the dashed line represents the atrial compliance. *LA* left atrium.

**Figure 12:**
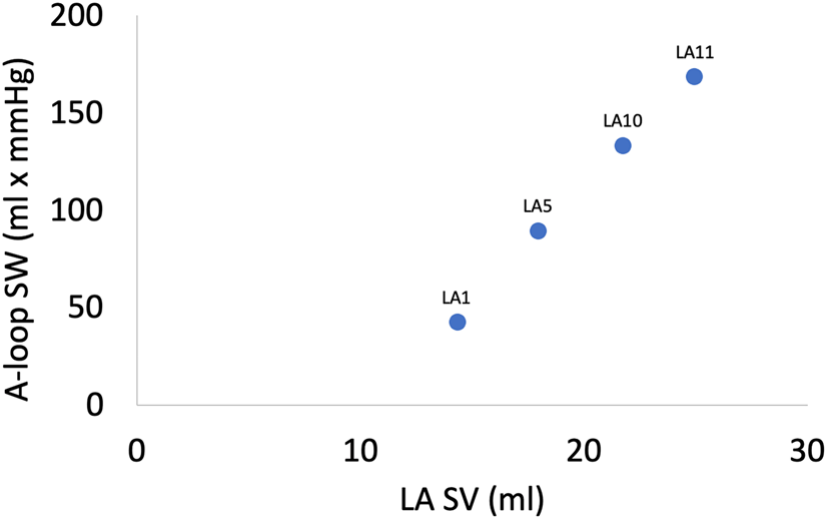
The relationship between the A-loop stroke work and left atrial stroke volume for LA1, LA5, LA10, and LA11. *LA* left atrium, *SW* stroke work, *SV* stroke volume.

The pressure–volume loops for LA1, LA5, LA10, and LA11 are shown in Fig. 11. The ratio between the outlet volume based on the MV flow recording and the inlet volume based on the flow recording on one of the four PVs is shown in Table 4 along with the stroke work of the A and V loop. Figure 12 depicts the relationship between the LA A loop stroke work and LA stroke volume.

**Table 4:** The ratio between the mitral valve outlet volume and inlet volume from one pulmonary vein, the left atrial stroke volume and stroke work of the A and V loop, respectively, the ratio between the stroke works, and the estimated compliance for the artificial left atrium number 1, 5, 10, and 11.

LA	MV/PV	LA SV (ml)	SW A (ml·mmHg) (mJ)	SW V (ml·mmHg) (mJ)	SW A/SW V	C (ml/mmHg)
1	3.55	14.34	42.14	247.43	0.17	2.00
			5.62	32.99		
5	5.41	17.96	88.94	173.79	0.51	2.10
			11.86	23.17		
10	4.28	21.75	132.85	297.64	0.45	2.52
			17.71	39.68		
11	3.82	24.95	168.17	211.61	0.79	2.95
			22.42	28.21		

*C* compliance, *LA* left atrium, *MV/PV* ratio between mitral valve outlet volume and pulmonary vein inlet volume over one heart cycle, *SV* stroke volume, *SW A* stroke work for the A loop, *SW V* stroke work for the V loop.

**Figure 13:**
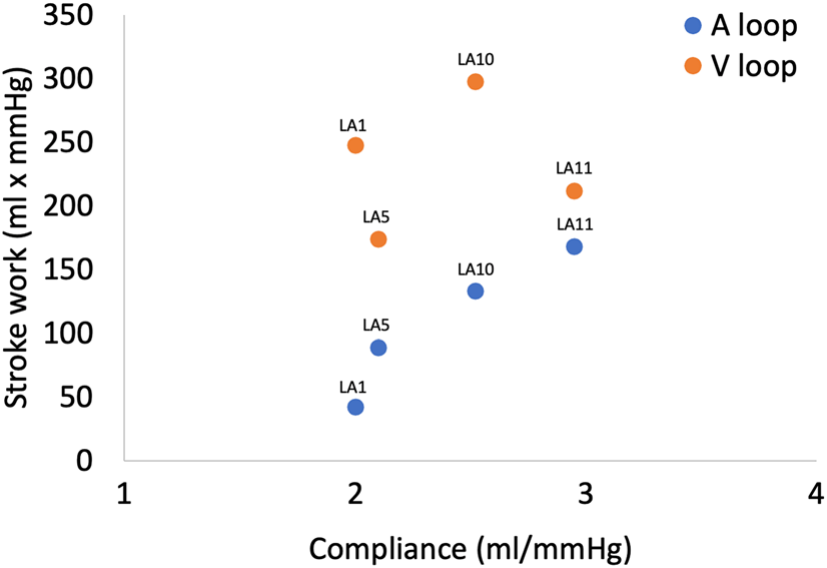
The stroke work of the A loop and V loop for the artificial left atrium (LA1, LA5, LA10, LA11) plotted as a function of the left atrial compliance. *LA* left atrium.

The estimated compliance from the pressure–volume loops is presented in Table 4. The stroke work of the A and V loop is plotted as a function of compliance for the four casted LAs in Fig. 13.

## Discussion

In this study, we have developed an in vitro MCL capable of simulating both normal and pathophysiologic conditions of the left side of the heart. The model comprises a versatile design that enables the precise adjustment and finetuning of parameters such as PV pressure, LA and LV phasic functions, and systemic arterial compliance and resistance.

### Left atrial compliance

The passive mechanical properties responsible for the behavior during the reservoir and conduit phase required a compliance similar to the native LA. To replicate physiological LA compliance, an expansion test was conducted using ex vivo porcine hearts as a reference. Although porcine hearts may exhibit different expansion characteristics than those of a living human heart, they are commonly used due to their similarities to the human heart^56^. The expansion test provided a measure of the overall mechanical properties of the LA but did not account for potential locally differences in tissue properties. However, due to the simplified geometry of the LA model, the same material and thickness were used to achieve the overall global biomechanical properties, while meeting these design constraints.

Different forms of silicone were used as the hyperelastic material for the LA model. This enabled the casting and creation of LA models with a compliance within the range observed in the ex vivo porcine heart experiments, as well as beyond this range. This feature represents the first characteristic of this in vitro model as a platform for investigating the effect and possible interventions of altered LA compliance. For example, this is relevant when investigating paroxysmal and permanent atrial fibrillation in heart failure with preserved ejection fraction, where a decrease in LA compliance caused by LA myopathy is observed during increased atrial fibrillation burden^57^.

The estimation of the compliance from the pressure–volume loops revealed an anticipated increase from LA1 to LA11, aligning with the expansions test results. However, estimating the compliance within the flow loop with the LA in situ may provide a better functional representation of the LA compliance. Nevertheless, this approach has limitations, as this average compliance estimate is based on a selected segment of the LA reservoir phase derived from the pressure–volume loops.

### Left atrial and left ventricular cycle control

Another important characteristic of this model is the ability to independently control the timing and duration of the diastole and systole in the LA and LV. This allows for the simulation of conduction disorders such as atrioventricular block and cardiac arrhythmias like atrial fibrillation. Patients with atrial fibrillation have a five-fold risk of stroke^58^, and the primary mechanism for stroke is the formation of stagnant flow in the LA and the LA appendage, leading to on-site thrombosis and embolization to the cerebral circulation. Expanding the MCL model to include a patient-specific geometry of the LA would allow for the investigation and characterization of LA flow patterns to identify areas of low flow zones and stagnation^59^ before and after LA appendage occlusion during both sinus rhythm and atrial fibrillation.

Moreover, since the LV volume is solely determined by the pump control signal, this in vitro MCL also has the capability of precisely adjusting the LV systolic and diastolic function. This feature allows for simulating conditions such as restrictive cardiomyopathy^60^.

### Fluid dynamic behavior

The measurements of the LA and LV pressure shown in Fig. 9E,F are in good agreement with the physiological data reported in the literature^21,22^. The v-wave peak in the LA pressure was found to be 16 mmHg, which is within the normal range of 6–21 mmHg^61^. This peak is influenced by factors such as LA compliance and LA filling. The a-wave peak in the LA pressure was measured to be 24 mmHg, which is higher than the range of 4–16 mmHg^55^. This increase in the LA pressure may be attributed to the contribution of the late diastolic portion of the piston waveform (Eq. (3)) to the LA contraction as well as the pressurization of the LA container during LA systole. The c-wave of the LA pressure is heavily influenced by the movement of the MV. However, in this in vitro model, the MV and the MV annulus were rigid, and therefore, only a small c-wave was observed.

Despite the absence of the isovolumetric contraction and relaxation phases due to the rigid LV chamber, all other components of a physiological LV pressure waveform were present in the measured signature shown in Fig. 9F. The peak systolic value of the LV pressure of 115 mmHg is within the normal range^62^. The synchronization between the LA and LV pressure, as seen in Fig. 9G, confirms the coordination of these events.

A key area of investigation was to understand the factors that determine the PV flow phases, which is crucial in designing the inlets of the LA. Some studies suggest the diastolic forward flow (the D-wave) to primarily be determined by LV relaxation and a drop in LA pressure due to MV opening^11,16,17,19,63^, while others suggest that it is primarily determined by the recoil of the LA, driven by stored elastic energy during the LA reservoir filling^6,13^. The retrograde PV flow, seen as the A-wave, has primarily been ascribed to the LA contraction^19,20^. The PV systolic forward flow (the S-wave) has been suggested to primarily be caused by LA related events during LV systole, such as LA relaxation, LA compliance, and MV annulus movement^6,13,16,63–65^. However, others suggest that it is driven by the right ventricular systolic pressure propagation through the pulmonary circulation^66–68^. The LA reservoir, LV, and right ventricular function has also been proposed as contributing factors^6,14^. In this study it was assumed that the PVs are isolated from the effects of the right side of the heart and solely influenced by events from the left side of the heart, and that the pulmonary capillary bed can be considered a reservoir with a large capacity^67^. Therefore, a rigid reservoir with constant static pressure was designed to create and maintain the PV pressure for the LA inlets.

The PV flow waveform recorded in vitro exhibited a similar pattern to the physiological waveforms recorded in vivo using magnetic resonance flow imaging and was characterized by the presence of distinct A-, S-, and D-waves. However, it was noted that the in vitro recordings displayed only a single S-wave peak, whereas the in vivo recordings displayed two peaks at the S-wave (S1 and S2), which also has been previously reported^11–14,16–19,69^. The S/D ratio observed in the in vitro PV flow was within the range considered normal^17^.

Only one PV flow rate was measured and used for normalizing the total PV inlet to the MV outlet. If the flow in the four PVs was completely symmetrical, this number could have been multiplied by four. However, the results showed that the inlet measured in one of the PV had to be scaled from 3.55 to 5.41 to match the MV outlet. This indicate that representing the total PV flow through the measurement of only one can cause some errors. The assumption of no leak in the in vitro flow loop may also be challenging to achieve.

### The impact of left atrial compliance on fluid dynamics

The pressure and flow waveforms recorded have been carefully optimized through adjustment of the various elements of the MCL. In particular, the role of the LA compliance has shown to be crucial for achieving recordings that resemble physiological conditions. Neglecting LA compliance or using improper compliance in designing MCLs may lead to conflicting results. For example, Mouret et al.^50^ found the measured in vitro PV flow results not to be comparable to the in vivo flow data, assumable due to a discrepancy in LA compliance.

In this work, in vitro experiments with four different levels of LA compliances were conducted (Fig. 10 and summarized in Table 3). The impact of LA compliance on PV flow (Fig. 10C) revealed that a decrease in LA compliance leads to a reduction in PV systolic forward flow (S-wave) and an increase in PV diastolic forward flow (D-wave), resulting in a lower S/D ratio. This shift in PV flow from predominantly systolic flow to predominantly early diastolic flow has also been reported to depend on LA compliance in vivo^17^. As LA compliance decreases, the retrograde PV flow of the A-wave shifts to become positive forward flow. Additionally, the valley between the S- and D-wave in late LV systole changes from forward to retrograde (negative). During the same time of the heart cycle, it is observed that the LA pressure (Fig. 10A) increases as LA compliance decreases. This increase in pressure is a consequence of the lower compliance, as compliance describes the relationship between volume change and pressure changes. With less compliance, the same volume change during the LA reservoir phase will result in an increase in LA pressure. However, as the LA becomes more restricted, it can accommodate less volume during the reservoir phase, and a new equilibrium is reached between the increase in pressure and the volume accommodated, resulting in the reduced S-wave. Since cardiac output was kept constant, a compensation was seen during the conduit phase where the D-wave increased. As a result, the pulmonary bed becomes part of the venous reservoir capacitance during the LV systole, and the increased LA pressure in this phase reverses the PV flow between the S- and D-wave.

A more restrictive (less compliant) LA makes it less compressible during the LA contraction. Therefore, even when pressurized, the reduced compression leads to less emptying, which could explain the change in the A-wave of the PV flow from negative to positive flow. However, by decreasing the compliance of the LA chamber, the model can be adjusted to mimic only impaired restrictive LA diastolic function and normal LA systolic function by increasing the systolic pressure of the LA container, which would increase the LA stroke volume.

The MV flow showed a reduction in the amplitude of both the E- and A-waves when LA compliance was decreased. This decrease in the A-wave during LA contraction may be caused by impaired LA systolic function, which also led to the change in the A-wave of the PV flow. The impaired LA contraction phase seems to functionally alter it toward becoming an extension of the LA conduit phase. At the same time, the E-wave of the MV flow is not only decreased but also vaguely broadened. As the cardiac output was kept constant and the leak of fluid from the MCL was minimal, it seems that the early diastolic filling may become slightly more dominant in the filling of the LV. The change in the E-peak may be explained by the change in LA compliance as the LV chamber is rigid and the same pulsatile pump waveform was applied in all cases.

The stroke work of the various LA models indicated that the active stroke work (A loop) rises with increased compliance while the passive stroke work (V loop) did not exhibit such pattern. With only four variations of LA compliance, caution in interpretating these results should be taken. However, a more compliant LA is likely to be more accommodating during the passive filling, potentially resulting in a larger stroke volume. Figure 12 illustrates a strong relationship between stroke volume and stroke work, supporting the above.

### Study limitations

The porcine hearts were acquired from a slaughterhouse and stored cold until preparation and conduction of the compliance tests. During the storage and transport the tissue could potentially have degraded. The expansion response recorded ex vivo was a result of passive tension, primarily governed by the extra cellular matrix (collagen) and titin. It may therefore represent a different response than in vivo.

In the MCL, mechanical heart valves were used and mounted in rigid holders. Even though they may provide proper valve function they are prone to cause flow disturbances and oscillations that are not seen in native valves. The rigid annulus structures of the valves also affected the response of the model, as described by the small c-waves in the atrial pressure recordings.

When simulating resting conditions for an average adult in vitro, cardiac output is typically set to around 5 l/min. In this study we used 4.5 l/min, which is 10% below this commonly used value. However, there is a large variation in the reference range of a normal cardiac output. In adults (ages 18–83) the cardiac output average and range (2 × SD) for women are reported to be 4.5 l/min (2.7–6.3) and 5.6 l/min (3.4–7.8) for men^70^.

Another limitation is that the model does not incorporate the impact of respiration on venous flow within the thoracic region. The PV pressure is merely controlled as a static pressure head. This is an issue that should be further investigated in future development of the system.

## Conclusion

This study presents a new versatile left heart MCL that accurately captures the complex and interrelated sequences of left heart events and provides supplementary understanding of left heart fluid dynamics. The results of the study demonstrated fluid dynamic characteristics with a highly physiological resemblance. Additionally, conducting measurements using LAs with different compliances allowed for investigation of the effects of LA compliance on left heart hemodynamics.

The findings suggest that the MCL has the potential to be used for investigating cardiac conditions and interventions related to LA-LV interactions, such as LA fibrillation, LA appendage occlusion, heart failure with preserved ejection fraction, LA myopathy and hypertrophy, MV insufficiency, MV repair, and AV conduction disorders.

## Data Availability

The datasets generated and analyzed during the current study are available from the corresponding author upon reasonable request.

## Abbreviations

LA: Left atrium
PV: Pulmonary vein/venous
LV: Left ventricle/ventricular
MV: Mitral valve
MCL: Mock circulatory loop
